# SAS-Pro: Simultaneous Residue Assignment and Structure Superposition for Protein Structure Alignment

**DOI:** 10.1371/journal.pone.0037493

**Published:** 2012-05-25

**Authors:** Shweta B. Shah, Nikolaos V. Sahinidis

**Affiliations:** 1 Department of Chemical Engineering, Carnegie Mellon University, Pittsburgh, Pennsylvania, United States of America; 2 Lane Center for Computational Biology, School of Computer Science, Carnegie Mellon University, Pittsburgh, Pennsylvania, United States of America; University of Cyprus, Cyprus

## Abstract

Protein structure alignment is the problem of determining an assignment between the amino-acid residues of two given proteins in a way that maximizes a measure of similarity between the two superimposed protein structures. By identifying geometric similarities, structure alignment algorithms provide critical insights into protein functional similarities. Existing structure alignment tools adopt a two-stage approach to structure alignment by decoupling and iterating between the assignment evaluation and structure superposition problems. We introduce a novel approach, SAS-Pro, which addresses the assignment evaluation and structure superposition simultaneously by formulating the alignment problem as a single bilevel optimization problem. The new formulation does not require the sequentiality constraints, thus generalizing the scope of the alignment methodology to include non-sequential protein alignments. We employ derivative-free optimization methodologies for searching for the global optimum of the highly nonlinear and non-differentiable RMSD function encountered in the proposed model. Alignments obtained with SAS-Pro have better RMSD values and larger lengths than those obtained from other alignment tools. For non-sequential alignment problems, SAS-Pro leads to alignments with high degree of similarity with known reference alignments. The source code of SAS-Pro is available for download at http://eudoxus.cheme.cmu.edu/saspro/SAS-Pro.html.

## Introduction

Protein alignment is a problem that has gained tremendous attention in bioinformatics and proteomics due to its applicability in protein clustering, identifying homology relationships, and inferring structure-activity relationships about new and existing proteins. Proteins may be compared with each other through sequence alignment, where the similarities between the proteins are identified through similarities within their amino acid residue sequences. Research on protein sequence alignment has led to the development of numerous dynamic programming algorithms [1], [2] that are central to the BLAST code [3], [4], an alignment tool that radically transformed the bioinformatics field and found extensive applications in the biotechnology industry. However, structural information of proteins is difficult to infer from sequence information alone. While sequence similarity generally implies structural similarity between proteins, there exist a large number of protein pairs, including haemoglobin and myoglobin found in the human body, that are structurally similar but possess low sequence similarities (also known as twilight zone proteins). Physical comparisons of protein structures [5], [6] further demonstrate the need for direct comparison of 3D protein structures, also known as the protein structure alignment problem, which is the focus of this paper.

The aim of protein structure alignment is to determine structural similarities between a given pair of proteins so that further functional relationships between them may be identified. Thus, protein structure alignment tools are useful in systematic classification of proteins based on their functional and homology relationships. They may be further employed in predicting functional properties of newly discovered or newly synthesized proteins based on structural similarity with existing proteins. Protein structure alignment tools may also be used in the pharmaceutical industry to determine alternative options for existing drugs, or development of personalized medication. Further applications are also possible in the bio-catalysis and other protein-based product industries, where structure alignment tools could help in development of new protein-based products.

Over the past three decades, a variety of algorithms have been developed for finding protein structural alignments, which has turned out to be a very difficult computational problem. Kolodny et al. [7], Gibrat et al. [8], Lancia and Istrail [9], Singh and Brutlag [10], and Novotny et al. [11] provide descriptions and comparisons of the most frequently used structure alignment tools. These tools include DALI [12], CE [13], Structal [14], and SSM [15], all of which are known to provide good quality sequential alignments in low computational times. These tools have been instrumental in the development of various protein structure databases like FSSP [16], SCOP [17], CATH [18] and HOMSTRAD [19], which provide extensive information on classification of protein folds and domains. However, these alignment tools employ heuristic methods and provide only approximate alignments with no guarantee of optimality. This may lead to inaccurate conclusions about relationships between proteins. Thus, for accurate analysis of structural similarities, exact structure alignment tools are required. Lancia et al. [20], Caprara et al. [21], Xie and Sahinidis [22], and Wohlers et al. [23] have developed exact structure alignment algorithms based on contact maps representations of proteins. However, these exact algorithms are often computationally expensive and may not be practical for performing a large number of structure comparisons. The development of protein structure alignment tools that strike a balance between fully optimal alignments and low computational requirements remains a challenge.

Early protein structure comparisons were based on computing the root mean square deviation (RMSD) amongst two protein structures of known residue correspondence. In order to make such comparisons on a large-scale, McLachlan [24] and Sippl [25] developed algorithms for fast RMSD computations. These algorithms were then used to construct the first protein structure alignment tools [26]–[28] that were based on determining the optimal correspondence amongst individual residues of two proteins. The structure alignment problem is traditionally formulated as a continuous optimization problem, where similar protein substructures are superimposed onto each other to evaluate structural similarity through RMSD calculation. Here, the proteins are represented using the 3D coordinates of all the C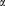 atoms representing the protein backbone. To obtain an alignment, one of the protein structures is rotated and translated to superimpose it onto the other protein structure, while optimizing a measure of similarity between them. Current structure alignment tools address the alignment optimization problem through a two-step process. In the first step, ‘assignment’ between amino-acid residues of two proteins is established using dynamic programming or heuristic methods. The objective here is to obtain the largest possible sequential alignment between the two proteins. In the second step, ‘superposition’ is achieved via computing optimal values for rotation-translation variables by various convex optimization techniques. In the superposition step, the RMSD value or a variant of the RMSD value is minimized. An iterative application of this process results in obtaining the final alignment. Structal [14], MAMMOTH [29], and alignment tools developed by Wu et al. [30], Andreani and Martinez [31], and Andreani et al. [32] are all based on this two-step approach. These approaches differ in the algorithms they use for assignment evaluation and structure superposition, as well as the choice of the objective functions in the two stages of alignment. Nearly all these methods determine the assignment by basic dynamic programming, and utilize different ways of building the similarity matrices based on different structural characteristics of the proteins. The exception is Andreani et al. [32], who determine the assignment of amino-acid residues by a heuristic method.

The two-step approach to structural alignment has clear computational advantages and results in very fast implementations. However, by decoupling the inter-dependence between the assignment and superposition problems, alignment tools based on this approach may produce suboptimal alignments. In this work, we present a novel approach, Simultaneous Alignment and Superposition of PROteins (SAS-Pro), that combines the evaluation of the assignment and the rotation-translation problems into a single bilevel optimization formulation. We further propose a combination of optimization algorithms, which we demonstrate leads to a practical computational approach for the solution of the proposed formulation.

By eliminating the residue-sequentiality constraints, the SAS-Pro approach is additionally capable of providing both sequential and non-sequential structure alignments. Most structure alignment tools developed in the past are designed to provide only sequential alignments between protein structures. However, there exist a multitude of similar protein pairs that exhibit non-sequential structure similarities. Thus, development of alignment tools to identify non-sequential similarities is important. This problem is only recently being addressed through the development of alignment tools such as STSA [33], and the introduction of non-sequential alignment capabilities in DALI [12] and SSM [15].

The remainder of this paper is structured as follows. After stating the protein structure alignment problem, the SAS-Pro optimization model is presented and a numerical solution algorithm is proposed. The implementation is subsequently discussed along with computational results, followed by conclusions.

## Methods

### The problem and a natural decomposition

Consider proteins A and B to be structurally aligned. Let 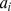 represent the 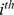 residue of protein A, and 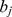 represent the 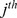 residue of protein B. In addition, let 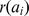 and 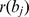 represent the 3D coordinates of the corresponding amino-acid residues. We seek to align amino-acid residues of A to amino-acid residues of B so that, when A is rotated-translated onto B, a similarity measure between the two proteins is minimized. The RMSD function will be used to determine the similarity between the protein structures and is defined as
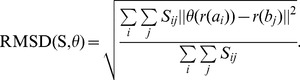
(1)Here, 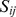 is a binary variable that equals 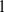 when 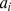 is aligned to 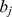 and 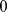 otherwise, and 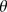 represents the rotation-translation transformation applied to protein A. The rotation-translation transformation is characterized by the three components of the translation vector and the 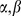, and 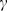 angles of rotations about the 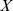, 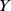 and 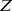 axes, respectively.

The problem of minimizing the RMSD may be represented as the following mixed-integer nonlinear optimization program:
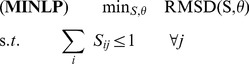
(2)

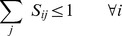
(3)

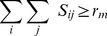
(4)

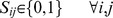
(5)Here, the parameter 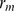 in Constraint (4) is the minimum number of residues that must be aligned to ensure that the global optimum of the model attains a non-trivial value. Constraints (2) and (3) ensure that no more than one amino-acid residue of protein A is aligned with an amino-acid residue of protein B and vice versa. Constraint (5) enforces the binary nature of the assignment variables 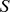.

#### Two-stage approach

A two-stage solution approach employed by existing alignment tools decouples the effects of 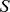 and 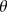 variables and evaluates the effect of the assignment variables 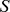 and rotation-translation variables 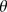 separately. The two-stage optimization problem may be viewed as follows:
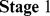


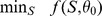


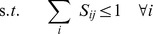


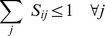


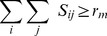
(6)

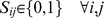


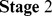


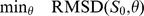
where 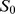 and 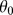 are optimal values of 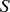 and 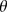, respectively, obtained in Stage 1 and Stage 2 of an iteration of the two-stage optimization problem. Constraint (6) in Stage 1 is imposed implicitly in the model by solution procedures utilized to solve for 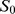.

In typical approaches, values for the assignment variables 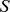 are determined by heuristic methods and dynamic programming techniques. The function 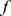 is thus selected as the dynamic programming objective function based on different similarity matrices designed for the alignment tool. The similarity matrices currently in use are based on structural features of the proteins, including inter-residue distances [14], [31], bond angles [29], and radii of fragment curvature [30]. These heuristic methods and dynamic programming techniques do not guarantee optimality of the alignment obtained with respect to the objective of Stage 2, the RMSD value. Thus, the final alignment obtained from the iterative procedure is not guaranteed to be globally optimal, and is known to be dependent on the initialization of the process [14], [31], [32]. Hence, the two-stage formulation may provide only a feasible solution of the MINLP and not necessarily a global optimum. Global optimality cannot be guaranteed unless the MINLP is somehow solved directly.

### SAS-Pro model

The SAS-Pro model reformulates the MINLP model into a single bilevel optimization problem. For any given 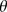, the function 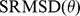 may be defined as
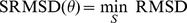



The master problem of the SAS-Pro model optimizes over the solution of the subproblem 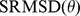. The bilevel SAS-Pro model is as follows:
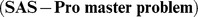


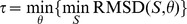


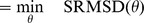


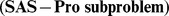


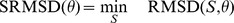


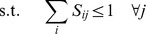


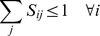


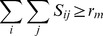


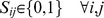
The master problem objective function 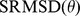 is in the space of the 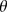 variables alone. Yet, it is trivial to see that any assignment/superposition feasible to the MINLP is also feasible to the SAS-Pro master problem. Hence, our reformulation maintains optimality.

Evaluation of the function 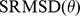 involves solving the subproblem and determining the optimal assignment variables 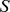, for given values of 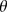 and parameter 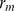. Our key observation is that, for a given value of 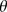, the subproblem can be reformulated as the following k-cardinality linear assignment problem (k-LAP):
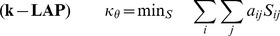
(7)

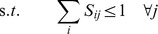


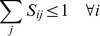


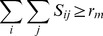
(8)

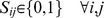
where 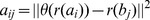, 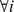, 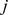. A highly efficient polynomial-time algorithm, SKAP [34], has been developed to solve the k-LAP problem and can be readily utilized in this context. The solution to the k-LAP problem will provide an assignment of exactly 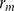 amino-acid residues, as constrained in equation (8). The numerical value of 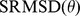 can be obtained from the objective value in equation (7) of the k-LAP problem as 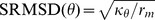. The k-LAP model does not include any sequence preserving constraints. Thus, the SAS-Pro model is designed to provide an optimal assignment and structure superposition of protein structures for specified values of the parameter 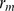, with no sequence-preserving constraints. We later show how to recover a sequential alignment, if desired, from the SAS-Pro alignment.

Kolodny and Linial [35] also present a bilevel approach to structure alignment by utilizing the SAS [36] similarity measure as the objective function in the master problem, as opposed to the RMSD value. They obtain values for the assignment variables 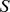 through a dynamic programming methodology and determine the rotation-translation variables by enumeration over a grid in the 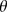 space. Our approach differs from their approach in three major aspects. First, the objective function used by Kolodny and Linial in the subproblem to determine the assignment variables 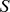 (dynamic programming based objective) differs from their master problem objective (SAS score). We use the same objective in both the subproblem as well as the master problem of the SAS-Pro model, which guarantees that a SAS-Pro optimal solution is optimal also for the original MINLP problem. Second, we utilize efficient search techniques to solve the master problem and obtain near-optimal rotation-translation variables, as opposed to the expensive enumeration approach used by Kolodny and Linial. Finally, our approach has the added capability of providing both sequential and non-sequential structure alignments for protein pairs.

As mentioned above, an optimal solution of the MINLP is feasible to our reformulation. In order for an optimal solution to be identified, suitable algorithms must be used to solve the master problem to global optimality. Indeed, there exist derivative-free optimization (DFO) algorithms that can achieve this goal based on dense sampling of the domain [37]. However, in the search of the most computationally efficient approach, in the next section we will also evaluate local search techniques for solving the master problem. With the same goal in mind, we will introduce a heuristic approach for determining the optimal parameter 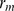 as well as for curtailing the number of degrees of freedom for the alignment problem.

### Algorithm

#### Derivative-free optimization

**Figure 1:**
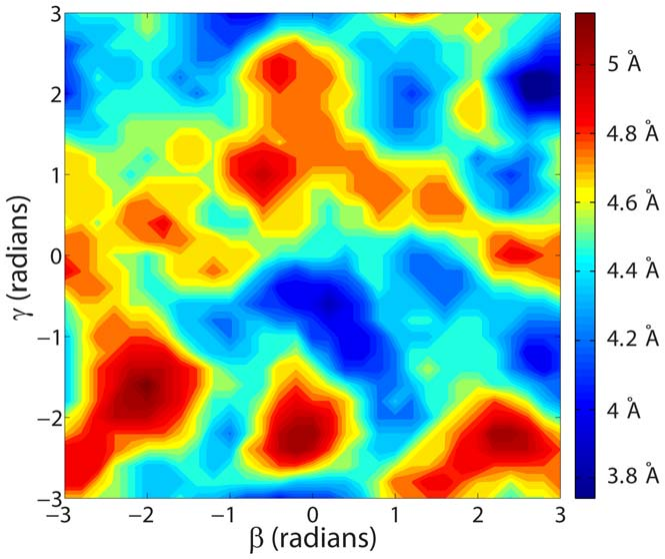
Contour plot of the landscape of the RMSD function for 1B00 and 1DBW proteins in the 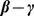 rotation angles plane.

The landscape of the RMSD function with varying values of the rotation angles 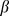 and 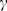 is presented in the contour plot of Figure 1 for proteins 1B00 and 1DBW. As seen in this figure, the objective function in the SAS-Pro model is highly multi-modal and nonlinear. This multi-modality can be addressed by optimization techniques that span the entire search space of the problem in the search for global optima. Furthermore, an explicit algebraic form for the SRMSD objective function for the master problem is not available, thus making it difficult to utilize derivative-based optimization methods. Thus, we opted to employ DFO techniques in order to solve the SAS-Pro model.

We performed extensive computational analysis with 28 different DFO solvers, based on a variety of techniques that included direct search, pattern search, surrogate management frameworks, domain partitioning methods, local search, global search, deterministic and stochastic algorithms [37]. Our experiments indicated that the derivative-free solver SNOBFIT [38] provides the best performance for a small number of function evaluations. This observation is consistent with the results reported in [37]. Keeping the number of function evaluations low was dictated by our desire to design an algorithm that would take no more than a few CPU minutes on a standard computer workstation for the alignment of protein pairs that are routinely analyzed nowadays.

Our interface to SNOBFIT is based on the ‘mydfo’ interface developed by Rios [39]. We have limited SNOBFIT to 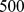 function evaluations for each value of the parameter 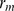. Every RMSD function evaluation for a given value of 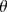 involves solving the k-LAP problem using the SKAP code developed by Dell'Amico and Martello [34].

#### Choice of parameter 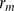


The solution to the SAS-Pro model is dependent on the parameter 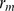. Different values of 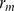 may lead to very different optimal alignments. The best alignment is found when the value of 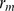 is close to the number of biologically relevant residue matches. It is therefore important to determine the right value of the parameter 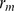. Furthermore, it is important for an implementation to select a value for this parameter *automatically*, i.e., without requiring the user to specify it. This is achieved here as follows.

Proteins with high level of similarity have a large length of alignment, usually corresponding to 85% or more of size of the smaller protein. Hence, the number of biologically relevant residues matches is expected to be between to 85% to 100% of the size of the smaller protein. To identify the best value for 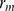, we systematically vary the value of 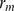 from 100% to 85% of the size of the smaller protein, until an alignment with a good similarity measure cutoff is obtained. The similarity measure used here is SAS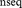, a modified version of the SAS score, that is further discussed later in this paper. In our implementation, for a given structure alignment problem, we evaluate structure alignments for different values of 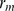 and select the one for which an SAS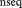 score of less than 4 Å is obtained.

Lower levels of similarities between proteins may arise while attempting to obtain a match of a smaller substructure from one protein with other proteins. In order to use SAS-Pro in such a context, it is advisable to isolate the relevant substructure in question before comparing with larger proteins. This will increase the chances of obtaining a suitable alignment within the limits of choice of the parameter 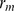.

#### Reducing the number of degrees of freedom

The solution to the SAS-Pro model involves determining the optimal values of both the assignment variables 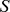 as well as the rotation-translation variables 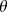. The assignment variables 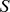 are obtained as an exact solution to the SAS-Pro subproblem. Thus, the only degrees of freedom available in the SAS-Pro master problem are the three translation vector components 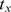, 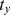, and 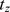 along the X, Y and Z axes, respectively, and the three rotation angles 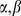, and 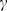 about the X, Y and Z axes, respectively.

In the course of our computational experimentations, we observed that, for proteins with similar sizes, a good approximation of the translation vectors is very often obtained if the centroids of the two protein structures are required to coincide. Thus, while comparing proteins of similar sizes, the number of degrees of freedom for optimization may be reduced to only the three rotation angles. As demonstrated in [37], for a collection of over 500 test problems, problems with up to three or four variables were almost always solved to global optimality by a variety of DFO algorithms. Thus, while solving the SAS-Pro optimization problem, the small number of degrees of freedom provides a computational advantage in terms of obtaining globally optimal structure alignments.

For structural comparison of proteins with different sizes, the SAS-Pro algorithm offers an option to utilize all six degrees of freedom. In this case, in order to maintain solution quality of the DFO solvers, we found it necessary to increase the number of function evaluations to 1000 for each value of 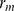 considered.

#### Extracting sequential alignments

The solution to the SAS-Pro model is usually a non-sequential structure alignment between the two proteins. However, a sequential alignment is easy to extract from the non-sequential alignment obtained from the SAS-Pro algorithm in a post-processing step. A dynamic programming algorithm was designed to identify the largest sequential alignment amongst the aligned residues provided by SAS-Pro. This algorithm sequentially evaluates the largest length of sequential alignment terminating at residue 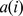 of protein A and stores it in the vector 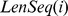. The algorithm maintains a pointer to the residue before 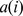 in the sequential alignment in the vector 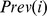. 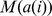 denotes the residue 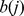 of protein B which is aligned to 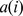. The largest value of 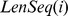 provides the length of the largest sequential alignment terminating at residue 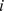. Backtracking the residues from this value of 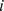 using the vector 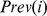 provides the corresponding alignment. A pseudo-code of the algorithm is presented below:




#### Similarity measure

For sequential protein alignments, where the sequence of the amino acid residues is preserved in the alignment, many suitable similarity measures, such as the Structure Alignment Score SAS [36] and the Similarity Index SI [40], have been defined. These measures are based on weighted ratios of the RMSD value and the length of alignment produced by the algorithm:
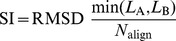
(9)

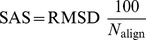
(10)Here, 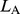 and 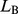 represent the lengths of the proteins A and B, and 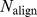 represents the number of sequentially aligned residues between the two proteins. For non-sequential structure alignments, the length of alignment is not properly defined and hence cannot be used to calculate the SAS and SI measures. We introduce a new measure of length of alignment, the total fragment length (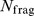), to extend the definition of the SAS similarity measure to non-sequential structure alignments. Following earlier works [41]–[43], the total fragment length is defined as the sum of lengths of aligned continuous fragments of five or more residues. Sequentiality of the amino-acid residues in the fragment is not required, thus providing for a measure of the length of alignment that is applicable to both sequential and non-sequential structure alignment.

The similarity between proteins is then determined using the proposed SAS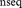 measure, which is defined as
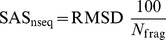
(11)This measure reduces to the SAS measure for the case of sequential structure alignments.

The best non-sequential structure alignment obtained from the SAS-Pro algorithm may include multiple local small-length matches as opposed to a single large global alignment. This disorder of the alignment can be measured by the value of the fragment length. A disordered alignment is expected to have a small fragment length, while a biologically relevant ordered alignment is expected to have a large fragment length, thus providing lower SAS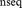 values for biologically relevant alignments. Hence, the best alignment for a given pair of proteins is expected to be one with the lowest SAS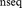 score.

## Results

We performed computational experiments based on three data sets:

- the Sokol data set [44], which is a set of 9 small size proteins with proteins from three different fold families,
- the Skolnick data set [20], which is a set of 40 large globular proteins from four different fold families from the SCOP data base, and
- the RIPC data set [45], which is a set of 23 complex structure alignment problems.

An all-to-all pairwise alignment for all the proteins in the Sokol and Skolnick data sets was obtained, resulting in 850 pairwise alignment problems with 222 similar protein pairs and 628 dissimilar protein pairs. The Sokol data set includes 20 similar protein pairs that align sequentially. The Skolnick data set consists of proteins from four fold families: a) Flavodoxin-like fold CheY-related, b) Plastocyanin, c) TIM beta/alpha-barrel, and d) Ferratin. Protein pairs within the same fold family are termed as similar pairs and exhibit sequential similarity. The RIPC data set consists of 23 protein alignment problems for which a biologically relevant reference alignment is available. These 23 alignment problems are complex and exhibit non-sequential structure similarities. The complexity of these alignments arises from repetitions, insertions/deletions, permutations, and conformational changes between the protein pairs that are not easily handled by alignment algorithms. All data sets are provided at http://eudoxus.cheme.cmu.edu/saspro/SAS-Pro.html.

In all tests, the typical computing time requirements for SAS-Pro were around 1 CPU minute per protein pair on an Intel Quad Core 2.83 GHz processor with 6 GB RAM, while providing sequential and non-sequential alignments with exceptional classification ability.

### Sequential structure alignments

The Sokol and Skolnick data sets were analyzed to evaluate the performance of SAS-Pro in obtaining sequential alignment problems. To obtain sequential alignments from the non-sequential alignments provided by SAS-Pro, the procedure described in the subsection entitled “Extracting sequential alignments” was used. Alignments were compared using the RMSD values as well as the geometric similarity measures SI and SAS.

A comparison of the RMSD, SI, and SAS values obtained by SAS-Pro for similar and dissimilar proteins is presented in Table 1. For protein pairs within the same fold family, alignments with low RMSD, SI, and SAS values were obtained. For pairs from different fold families, the values of RMSD, SI, and SAS were comparatively higher than the corresponding values for similar proteins. In addition, the alignments obtained from the SAS-Pro alignment tool were near-sequential for similar protein pairs and were 96% in agreement with known optimal alignments between the proteins that were obtained from the exact structure alignment tool CMOS [22]. These optimal alignments contain both large fragments of aligned residues as well as a few isolated aligned residues. SAS-Pro matches the large fragments of aligned residues with these optimal alignments exactly. However, the alignments may differ in isolated residue matches, that are not of biological consequence, resulting in an average of 96% agreement between the alignments between SAS-Pro and CMOS.

**Table 1:** Average (standard deviation) RMSD value, SI score, SAS score, and match with reference alignments for the Sokol and Skolnick data sets for similar and dissimilar protein pairs.

	Sokol set		Skolnick set	
	Similar	Dissimilar	Similar	Dissimilar
RMSD	0.60 (0.4)	2.9 (1.45)	1.72 (0.78)	3.94 (0.6)
SI	1.17 (0.4)	7.04 (1.45)	3.15 (1.23)	9.77 (3.9)
SAS	1.61 (0.7)	7.37 (1.78)	2.19 (0.89)	8.51 (2.9)
% agreement with optimal alignment	96	N.A.	96	N.A.

The alignments obtained from SAS-Pro were also compared with those obtained from the CE [13], SSM [15], and STSA [33] alignment tools. Raw comparison results for SAS-Pro and other methods are provided in File S1.zip of the Supporting Information. The results are summarized in Table 2. The SAS-Pro approach provided alignments with better or equal RMSD for over 59 to 69% of the similar structures. For some problems, SAS-Pro was able to provide RMSD, SI, and SAS scores which were smaller by more than 4 Å than those obtained from CE. Moreover, the RMSD values of more than three quarters of the remaining problems were observed to exceed those in CE and SSM by only a single standard deviation (0.5 Å), while preserving a 96% similarity with the corresponding sequential structure alignments. Consequently, the corresponding SI and SAS scores for these problems were also within a single standard deviation of those from CE and SSM. t-test results for SAS-Pro, CE, SSM, and STSA show that these algorithms distinguish between similar and dissimilar protein pairs with the same high significance (t-test value 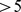). However, SAS-Pro has lower mean and standard deviation values for the similarity measures, resulting in better quality solutions with an average t-test significance value of 0.5.

**Table 2:** Comparison of SAS-Pro with CE, SSM, and STSA for the similar protein pairs of the Sokol and Skolnick data sets using RMSD, SI, and SAS measures.

	% Problems where					
	SAS-Pro is better			SAS-Pro is at par		
Solver	RMSD	SI	SAS	RMSD	SI	SAS
CE	57	51	51	12	12	12
SSM	47	36	36	12	12	12
STSA	44	40	40	21	21	21
	Average (standard deviation) improvement obtained by SAS-Pro (Å)					
Solver	RMSD	SI	SAS	RMSD	SI	SAS
CE	0.45 (0.46)	0.3 (0.41)	0.3 (0.42)	N.A.	N.A.	N.A.
SSM	0.26 (0.2)	0.2 (0.12)	0.16 (0.1)	N.A.	N.A.	N.A.
STSA	0.4 (0.15)	0.4 (0.15)	0.21 (0.1)	N.A.	N.A.	N.A.

The table presents the percentage of problems where SAS-Pro performed better than, or at par with CE, SSM, and STSA. In addition, the table presents the average improvement in the RMSD, SI, SAS scores for these problems when SAS-Pro is used instead of other solvers.

The Sokol and Skolnick data sets together include 222 similar protein pairs and 628 dissimilar protein pairs. A classification of these 850 problems into similar and dissimilar pairs was sought based on the SAS scores of the alignments obtained. The CE, SSM, and SAS-Pro alignment tools provided exact classification of these protein pairs. The STSA algorithm, however, produced very short alignments for 5 of the similar pairs, leading to an imperfect classification.

**Figure 2:**
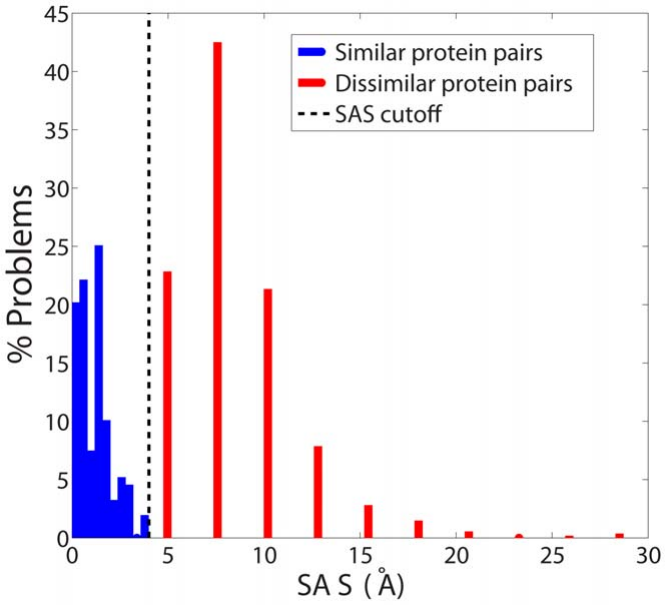
Distribution of SAS values obtained by SAS-Pro for similar and dissimilar proteins in the Skolnick data set. The means (standard deviations) for the similar and dissimilar protein pairs are 2.19 (0.89) and 8.51 (2.9) Å, respectively.

Figure 2 shows the distributions of the SAS values obtained for similar and dissimilar protein pairs for the Skolnick data set by SAS-Pro. The distributions for the similar and dissimilar proteins were observed to be completely disjoint, with lower SAS scores for similar proteins and higher SAS scores for dissimilar proteins. A SAS score cutoff of 4 Å produced a perfect classification of the alignment problems into similar and dissimilar protein pairs. Based on this observation, a termination criterion for the SAS-Pro code was implemented. For computations reported in the sequel, SAS-Pro was designed to terminate if (a) an alignment with a SAS score of 4 Å or less is obtained, or (b) all values of 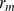 between 85% and 100% of the size of the smaller protein are explored. In either case, the best alignment and the corresponding RMSD, SAS score, and fragment length of the alignment are returned by the software.

### Non-sequential structure alignments

We performed a computational study to determine the quality of SAS-Pro's non-sequential structure alignments utilizing the RIPC data set and the non-sequential alignment problems presented by Salem and Zaki [33]. Salem and Zaki [33] provided two examples of non-sequential structure alignments for which their alignment tool, STSA, performs better than other structure alignment tools. We performed an alignment of the corresponding two protein pairs, 2LH3:A with 2HPD:A, and 1FSF:A with 1IG0:A, and obtained better alignments with SAS-Pro than STSA for both cases. For the 2LH3:A and 2HPD:A proteins, SAS-Pro provided an alignment with length 126 and RMSD 3.17 Å, as compared to STSA's alignment of length 117 and RMSD 3.27 Å. For the 1FSF:A and 1IG0:A proteins, SAS-Pro obtained an alignment with length 117 and RMSD 2.68 Å, as compared to STSA's alignment of length 104 and RMSD 5.4 Å. We present a quantitative comparison of the SAS-Pro alignment between the 2LH3:A and 2HPD:A proteins and other solvers in Table 3. As the results in this table demonstrate, SAS-Pro provides an RMSD in the same ball-park range as most other tools but with larger alignment length, thus providing a superior structure alignment as the SAS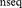 values indicate.

**Table 3:** Comparison of performance of alignment tools for aligning 2LH3:A and 2HPD:A proteins.

Alignment tool	RMSD (Å)	N 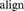	SAS 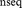
SAS-Pro	3.17	126	2.5
SARF2	3.05	108	2.8
STSA	3.37	117	2.9
STRUCTAL	2.27	56	4
CE	4.05	91	4.4
DALI	4.8	87	5.5

(All results, except SAS-Pro, taken from [33].)

**Figure 3:**
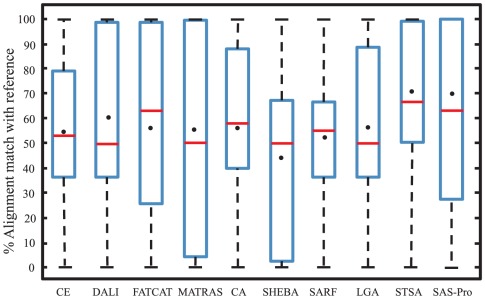
Box and whisker plot for the performance of different alignment tools for the RIPC data set. The red line represents the mean and the dot represents the median of the box. (All results, except for SAS-Pro and CE, were taken from [33]).

We next present results from a computational study with the 23 protein pairs in the RIPC data set. The 3D coordinates of the C-alpha atoms for the SAS-Pro alignments for the 23 pairs are provided in File S2.zip of the Supporting Information. For this test set, SAS-Pro provided alignments which are 30% to 100% in agreement with the reference alignments. The mean agreement of SAS-Pro is 62% and the median is 70%. SAS-Pro provides alignments with greater mean and median agreements than CE, DALI, FATCAT, MATRAS, CA, SHEBA, SARF, and LGA. The corresponding box and whisker plot of percentage agreement with reference alignments is shown in Figure 3. STSA provides alignments with better mean and median agreements with reference alignments than SAS-Pro. However, SAS-Pro provides excellent quality alignments with 100% agreement with the reference alignments for eight problems, while STSA provides alignments in 100% agreement with reference alignments for only four problems. Amongst the remaining alignment methods, only DALI, FATCAT, and MATRAS provide some (fewer than four) alignments that are in 100% agreement with the reference alignments. Even though STSA provides non-sequential alignments, it is bound by the sequentiality and choice of the five-residue fragments it utilizes. SAS-Pro is more flexible in allowing non-sequentiality, thus resulting in better alignments than STSA and other solvers for several problems. As suggested by Mayr et al. [45], while the provided reference alignments are biologically relevant, multiple alternative alignments that result in equivalent structurally optimal solutions may exist, especially for proteins with conformational variability and multiple insertions/deletions. In these cases, results obtained from different alignment tools may differ considerably, where one of the alignments matches with the provided reference alignment while others provide alternative optimal alignments.

**Figure 4:**
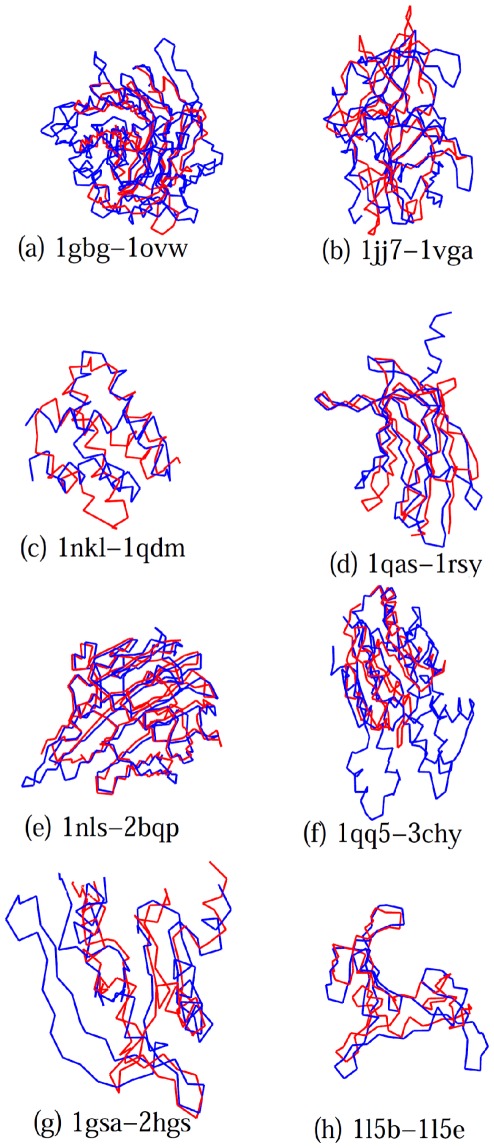
Alignments obtained by SAS-Pro for the RIPC data set. These alignments are in 100% agreement with the reference alignments [45].

The eight alignments for which SAS-Pro is in complete agreement with reference structures are shown in Figure 4. These eight protein pairs represent alignment problems spanning all four types of alignment challenges encountered in the RIPC data set, namely, repetitions, insertions/deletions, permutations, and conformational changes. The protein pairs 1gbg-1ovw (Figure 4(a)) and 1jj7-1vga (Figure 4(b)) present alignments with large requirements of insertions/deletions, not handled by all alignment tools. Specifically, 1gbg-1ovw are glucan hydrolase proteins with 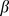-sandwich structure, while proteins 1jj7-1vga are P-loop containing NTP hydrolases that vary in the number of 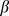-strands in the central region. Thus, these protein alignment problems require a large number of insertions/deletions for a good alignment. Mayr et al. [45] indicate that different alignment tools provide very different alignments for these proteins, usually matching only the N-terminal ADP binding site of 1jj7-1vga proteins correctly. SAS-Pro places no limit on the number of insertions/deletions, resulting in a very good alignment for these proteins. Protein pairs 1nkl-1qdm (Figure 4(c)), 1qas-1rsy (Figure 4(d)), 1nls-2bqp (Figure 4(e)), and 1qq5-3chy (Figure 4(f)) are examples of proteins with permutations. The 1nkl-1qdm, 1qas-1rsy, and 1qq5-3chy proteins consist of multiple 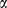-helices, which do not align sequentially. Most structure alignment tools mentioned above align the 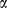-helices sequentially, resulting in incorrect structure alignments for these proteins. SAS-Pro correctly aligns the right 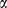-helices with each other, producing biologically relevant alignments. The 1nls-2bqp proteins have a 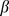-sandwich structure, where 1nls is posttranslationally cleaved, resulting in different N- and C-terminals. As a result, in the 1nls-2bqp protein pair, the N-terminus of one protein aligns with the C-terminus of the other protein and *vice versa*. Most alignment codes match only the N-terminus half of 1nls with the C-terminus half of 1bqp. Additionally, most alignment methods align only five out of the six reference alignment points, while SAS-Pro aligns the entire protein accurately. Finally, protein pairs 1gsa-2hgs (Figure 4(g)) and 1l5b-1l5e (Figure 4(h)) present conformational changes which cause slight bends in the structures. The 1gsa-2hgs proteins both contain the three-layered 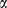-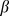-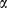 sandwich structure, similar to the Rossmann fold, while 1l5b-1l5e are both HIV-inactivating proteins with 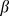-roll structures. SAS-Pro was able to provide the correct structural alignment with 100% match with the reference.

There are three problems in the RIPC data set for which the agreement of the SAS-Pro alignment with the reference in less than 50%. These three problems are from the permutation class of alignments for which, as Mayr et al. [45] suggest, biologically relevant alternative alignments may exist. Hence, it is likely that SAS-Pro's performance may be even better than what the results of this section suggest.

Mayr et al. [45] and Salem and Zaki [33] have discussed eight protein pairs from the RIPC data set that are difficult to align. Amongst these, Salem and Zaki [33] reported the 1nkl-1qdm protein pair and the 1qq5-3chy protein pair, for which most alignment tools provided a 0% match with the reference alignment. For both of these pairs, SAS-Pro and STSA provided a 100% match with the reference alignment. Amongst the remaining six protein pairs, SAS-Pro provided high quality alignments with 100% agreement with the reference for three pairs and over 50% agreement with the reference for the remaining three pairs.

## Discussion

In this paper, we presented a novel formulation of the protein structure alignment problem as a single bilevel optimization problem that addresses the assignment of amino acid residues and the structural superposition of proteins simultaneously. We employed derivative-free optimization techniques to deal with the multi-modality and non-differentiability of the RMSD function in the proposed formulation. The proposed structure alignment methodology is capable of providing both sequential and non-sequential alignments.

Our computational experiments demonstrate that the SAS-Pro model captures similarities within proteins accurately and provides alignments with lower RMSD values and larger lengths of alignments as compared to CE, SSM, and STSA for a majority of problems in the Sokol and Skolnick data sets. Moreover, SAS-Pro exhibits very good performance for the RIPC data set, for which it provided alignments with 100% agreement with the reference for a large number of protein pairs.

While the present methodology addresses both sequential and non-sequential alignments, future work should investigate the introduction of additional degrees of freedom (bond rotation) for the development of a more comprehensive structure alignment tool.

## Supporting Information

File S1. Results with the Skolnick-Sokol dataset.
(ZIP)

File S2. Results with the RIPC dataset.
(ZIP)
